# Genetic predisposition to adiposity, and type 2 diabetes: the role of lifestyle and phenotypic adiposity

**DOI:** 10.1093/ejendo/lvaf084

**Published:** 2025-05-02

**Authors:** Mengrong Zhang, Joey Ward, Rona J Strawbridge, Jana J Anderson, Carlos Celis-Morales, Jill P Pell, Frederick K Ho, Donald M Lyall

**Affiliations:** School of Health and Wellbeing, University of Glasgow, Glasgow G12 8TB, United Kingdom; School of Health and Wellbeing, University of Glasgow, Glasgow G12 8TB, United Kingdom; School of Health and Wellbeing, University of Glasgow, Glasgow G12 8TB, United Kingdom; Department of Medicine Solna, Karolinska Institute, Stockholm 17177, Sweden; School of Health and Wellbeing, University of Glasgow, Glasgow G12 8TB, United Kingdom; School of Health and Wellbeing, University of Glasgow, Glasgow G12 8TB, United Kingdom; School of Cardiovascular and Metabolic Health, University of Glasgow, Glasgow G12 8TA, United Kingdom; Human Performance Lab, Education, Physical Activity, and Health Research Unit, Universidad Católica del Maule, Talca 115 3745, Chile; Centro de Investigación en Medicina de Altura (CEIMA), Universidad Arturo Prat, Iquique 1100012, Chile; School of Health and Wellbeing, University of Glasgow, Glasgow G12 8TB, United Kingdom; School of Health and Wellbeing, University of Glasgow, Glasgow G12 8TB, United Kingdom; School of Health and Wellbeing, University of Glasgow, Glasgow G12 8TB, United Kingdom

**Keywords:** T2D, obesity, central obesity, BMI-PRS, WHR-PRS, lifestyle factors, adiposity

## Abstract

**Aims:**

Genetic predisposition to adiposity is associated with type 2 diabetes (T2D), even in the absence of phenotypic adiposity (obesity and central obesity). We aimed to quantify the overall contribution of obesity and modifiable lifestyle factors to the association between genetic predisposition to adiposity and the development of T2D.

**Methods:**

This prospective cohort study involved 220 703 White British participants from the UK Biobank. It examined the associations between genetic predisposition to adiposity [body mass index polygenic risk (BMI-PRS) and waist–hip ratio polygenic risk (WHR-PRS)] and incident T2D, as well as interactions and mediation via lifestyle factors (diet quality, physical activity levels, total energy intake, sleep duration, and smoking and alcohol intake) and phenotypic adiposity.

**Results:**

People with high phenotypic adiposity and high adiposity PRS values (>1 SD above the mean) had the highest risk of incident T2D (versus non-obese/central obese and non-high PRS). This was the case for BMI-PRS [hazard ratio (HR) = 3.72] and WHR-PRS (HR = 4.17). Lifestyle factors explained 30.5% of the BMI-PRS/T2D association (2.0% mediation; 28.5% effect modification), and lifestyle and obesity together explained 92.1% (78.8% mediation; 13.3% effect modification). Lifestyle factors explained 20.4% of the WHR-PRS/T2D association (3.4% mediation; 17.0% effect modification), and lifestyle and central obesity together explained 72.8% (41.1% mediation; 31.7% effect modification).

**Conclusions:**

Whilst phenotypic adiposity explains a large proportion of the association between BMI-PRS/WHR-PRS and T2D, modifiable lifestyle factors also make contributions. Promoting healthy lifestyles among people prone to adiposity is important in reducing the global burden of T2D.

**Significance**

In this large prospective study, we showed that high genetic predisposition to adiposity increased type 2 diabetes (T2D) risk, but lifestyle patterns can modify and mediate this effect. By focusing on obesity-specific polygenic risk (body mass index polygenic risk/waist–hip ratio polygenic risk), our novel approach of 4-way decomposition analyses revealed direct and indirect pathways through which genetic risk interacts with lifestyle. We quantified how diet, physical activity, and other modifiable lifestyle/adiposity risk factors could mitigate diabetes risk, offering actionable insights for clinicians. Individuals with high genetic risk and poor lifestyle choices on-average face the greatest risk, but targeted interventions could reduce this. These findings emphasized the importance of combining genetic risk with tailored lifestyle strategies to prevent and manage the risk of T2D.

## Introduction

Over the past 3 decades, the prevalence of type 2 diabetes (T2D) has increased greatly; it is now above 9% in adults globally and over 13% in specific regions such as the Americas, Middle East, and Pacific Island communities.^1^ Interventions to reverse this trend are essential to reduce the burden on individuals and health services. Type 2 diabetes onset is associated with a wide range of genetic, lifestyle, and environmental factors.^2^ Adiposity is a well-established risk factor for developing T2D through mechanisms including β-cell dysfunction and reduced insulin production.^3^ However, a meta-analysis^4^ and a cross-biobank analysis^5^ demonstrated that genetic predisposition to obesity was associated with the development of T2D, even after adjustment for body mass index (BMI)—suggesting that other mechanisms play a role.

A range of lifestyle factors—including physical activity, diet quality, energy intake, alcohol consumption, smoking, and sleep duration, are known to be associated with developing T2D.^6,7^ However, it is currently unclear the extent to which they contribute to the relationship between genetic predisposition to adiposity and development of T2D, either via or independently of phenotypic adiposity.

Whilst genetic predisposition cannot be modified, lifestyle factors can. Therefore, identifying lifestyle factors that mediate the association between genetic predisposition and T2D is important in reducing the risk of developing the condition. If lifestyle moderates the association between genetic predisposition and T2D, genetic predisposition could be used to target public health interventions at those most likely to benefit.^8^ However, existing studies investigating gene–lifestyle interactions and T2D have largely focused on single to relatively few T2D single-nucleotide polymorphism (SNPs)^6,9-11^ rather than the cumulative effect of polygenic predisposition to adiposity. There is evidence that genetic risk factors for respective outcomes may influence intermediate modifiable lifestyle factors,^12^ and we are not aware of this approach being taken for T2D.

In this study, we systematically analysed the extent to which lifestyle factors (physical activity level, energy intake, diet quality, sleep duration, smoking, alcohol consumption, obesity, and central obesity) modified the relationship between BMI and waist–hip ratio (WHR) polygenic risk scores (PRSs), and incident T2D. We focused on incident outcomes because prevalence may include more confounding where the outcome influences predictors. We also conducted mediation analyses to quantify the extent to which the pathway from genetic predisposition to adiposity and T2D was direct (independent of lifestyle) or indirect (genes predispose to lifestyle which predisposes to adiposity).

## Methods

### Population

This study used data from the UK Biobank which recruited participants from the general population, aged between 40 and 70 years.^13^ From April 2006 to December 2010, *n* = 502 536 participants attended one of 22 assessment centres across England, Wales, and Scotland, at which they completed a touch-screen questionnaire (including self-reported physical activity level, dietary intake, sleep duration, smoking frequency, and alcohol consumption), underwent physical measurements, and provided biological samples, as described elsewhere.^14^

The primary exposures in this study were BMI-PRS^15^ and WHR-PRS.^16^ The outcome was incident cases of T2D. Lifestyle factors (physical activity level, total energy intake, diet quality, sleep duration, frequency of alcohol consumption, and smoking status) and phenotypic adiposity (obesity and central obesity) were investigated as potential mediators and effect modifiers. Sex, age, sociodemographic deprivation, 10 genetic principal components, and genotypic chip at baseline were considered potential confounders and included as covariates in the statistical models.

Participants who self-reported non-White British ethnicity were excluded from the study to avoid genetic heterogeneity (being >90% of the sample), along with those whose BMI was <18.5 kg/m^2^ to avoid non-linear associations with BMI and reverse causation. We also excluded participants with prevalent and diagnosed T2D at baseline, those who had Hba1c levels >48 mmol/mol, taking metformin at baseline, those who reported never drinking alcohol because of potential confounding (eg, stopped due to poor health), those with missing data on BMI-PRS and WHR-PRS, those who failed genetic quality control, and those with missing data on physical activity level, diet quality score, alcohol consumption, sleep duration, and smoking status. The final study sample was *n* = 220 703. Due to the limited sample size for total energy intake (*n* = 101 439), participants with missing values on this variable were excluded only when investigating effect of the “total energy intake” lifestyle risk factor in analyses. UK Biobank received ethical approval from the North-West Multi-centre Research Ethics Committee (reference no. 11/NW/03820). All participants of UK Biobank provided written informed consent based on the principles of the Declaration of Helsinki before enrolment. This project was completed using UK Biobank data application 71392.

### Exposures

Genetic data were available for 488 377 participants.^17^ Of a total of 220 949 participants included in our study, 90% of samples were genotyped using Affymetrix UK Biobank Axiom Array with 825 927 markers (Santa Clara, CA, USA), and the remaining 10% were genotyped using the Affymetrix UK BiLEVE Axiom array with 807 411 markers. These 2 arrays are extremely similar, sharing more than 95% of the same content. Further information on the genotyping process is available on the UK Biobank website (http://www.ukbiobank.ac.uk/scientists-3/genetic-data), which includes detailed technical documentation.^17^

All quality control and genetic variables were derived by UK Biobank. A standard set of sample quality control procedures was used. Applying statistical tests designed mainly to check for consistency of genotype calling across experimental factors and the indicators of missing rate and heterozygosity to identify poor quality samples, conducting quality control specific to the sex chromosomes using a set of high-quality markers on the X and Y chromosomes.^17^ Markers present on both the UK BiLEVE and UK Biobank Axiom arrays were only used and those markers that failed to pass quality control in more than one batch, had a greater than 5% overall missing rate, and had <0.0001 minor allele frequency were excluded. Samples that were identified as outliers for heterozygosity and missing rate were removed.^17^

LDpred^18^ was used to generate the weighted BMI-PRS^19^ and WHR-PRS.^16^ LDpred accounts for linkage disequilibrium (LD) between SNPs, creating a single genome-wide score using an infinitesimal model. The raw summary statistics were adjusted using 1000 unrelated UK Biobank participants as the LD reference panel, who were not used in the main analyses. Scores were then generated using these LD-adjusted summary statistics in those who pass the same genetic quality control as above and were not used in the LD reference panel.

### Outcomes

The primary endpoint of this study was incident T2D, which was ascertained via linkage to hospital inpatient records and death registry which were available up to October 2022 for England; August, 31, 2022 for Scottish Morbidity Record; and May, 31, 2022 for Wales. Detailed linkage procedures can be found at the UK Biobank online resource (http://www.ukbiobank.ac.uk/). Type 2 diabetes was defined as an ICD-10 code E11 contained in a hospital admission or death record.

### Covariates

Area-based deprivation was measured by the Townsend score, which was derived from census data on housing, employment, social class, and car availability by postcode of residence.^20^ A higher Townsend score represents a higher level of deprivation. More detailed information can be found in the UK Biobank online protocol (http://www.ukbiobank.ac.uk).

Ten genetic principal components were adjusted for ancestral stratification derived by UK Biobank central team.^21^ Population stratification would be a confounder variable which occurs when there are existing ancestry differences in allele frequencies between individuals. The SNP chip used was included as a potential confounder as each has its own specific properties and performance in genotyping variants.^22^

Participants reported physical activity level by using the short form of the International Physical Activity Questionnaire, and low physical activity level was defined as <600 MET-min/week.^23^ Dietary information was reported by using the Oxford WebQ questionnaire^24^ based on the 7th edition of McCance and Widdowson's The Composition of Foods for total energy intake.^25^ High total energy intake was defined as >2000 kcal/day for women and >2 500 kcal/day for men, in accordance with NHS guideline (https://www.nhs.uk/live-well/healthy-weight/managing-your-weight/understanding-calories/). Food frequency questionnaire was used to generate dietary quality score^26^ for dietary quality. We included 9 food items (processed meat, red meat, total fish, milk, spread type, cereal intake, salt added to food, water, and fruits and vegetables). Participants’ scores were summated, with a minimum score of 0 representing the least healthy diet and a maximum score of 9 representing the healthiest diet. Low diet quality was defined as a diet quality score <5. The sample size for analyses using energy intake variables was 95 437. Smoking status was self-reported at baseline and classified as either ever smoker (current or former smoker) or never smoker. Alcohol intake was self-reported as the number of units consumed per week, and >14 units/week was defined as high alcohol intake. Self-reported sleep duration was categorized into abnormal sleep duration (<7 or >9 h/day) and normal sleep duration (7-9 h/day).^27^

During the baseline assessment, participants’ height (Seca 202 stadiometer; Sca) and weight (Bc-418 MA body composition analyzer; Tanita Corp) were measured by trained nurses.^28^ Waist and hip circumference was measured using a Wessex non-stretchable sprung tape measure.^29^ Body mass index was calculated from weight in kilograms divided by height in metres squared, and WHR was calculated as waist measurement divided by hip measurement.^30^ Obesity was defined as BMI ≥30 kg/m^2^. Central obesity was defined as WHR ≥0.85 in women and WHR ≥0.90 in men.^31^

### Statistical analyses

Participant characteristics were firstly compared by weighted BMI-PRS and WHR-PRS categories. Each PRS was transformed into z scores and categorized as: PRS < −1 (>1 SD below the mean), −1 ≤ PRS < 0, 0 ≤ PRS < 1, and PRS ≥ 1 in this analysis. All the lifestyle factors and phenotypic adiposity were classified as binary variables in the pre-specified deleterious direction. The sociodemographic and lifestyle characteristics were summarized by PRS category using percentages and compared using χ^2^ tests.

Firstly, we tested for statistical interactions between sex with BMI-PRS and WHR-PRS. Where the interactions were significant, the models were stratified by sex. Cox proportional hazard models were applied to the association between BMI-PRS/WHR-PRS and T2D adjusting for potential confounders (age, sex, and deprivation, multimorbidity count, 10 genetic principal components, and genotyping chip) (Table S1). Each of the lifestyle factors and phenotypic adiposity were then added sequentially.

The second set of analyses focused on interactions between PRS, lifestyle, and phenotypic adiposity. Separate Cox proportional hazard models were run for each lifestyle and adiposity factor. Multiplicative interactions were tested by including, in the models, interaction terms between individual lifestyle, adiposity factors, and obesity-related PRS (low PRS defined as PRS < 0; high PRS defined as ≥0), and relative excess risk (of T2D) due to interaction (RERI) was derived to determine whether interactions exceeded additive effects.^32^ Dichotomization of PRS was required to calculate RERI and only used for these analyses.

Finally, 4-way decomposition was used to quantify how much of the total association between BMI-PRS/WHR-PRS and T2D could be attributed to just mediation, just interaction, both mediation and interaction, and neither mediation nor interaction (direct effect).^33^ Because the Cox proportional hazard model operates on the logistic scale, it could be interpreted as: HR_overall_ = HR_interaction_ * HR_mediation_ * HR_direct effect_. The results were presented as the overall proportion of excess T2D cases attributable to additive interaction ([HR_interaction_ − 1]/[HR_overall_ − 1]) and mediation ([HR_mediation_ − 1]/[HR_overall_ − 1]). An overall model was also fitted to include all lifestyle factors (± adiposity) and adjusting for age, sex, deprivation, multimorbidity count, 10 principal genetic components, and chip. Total energy intake was not included in this overall model because of its smaller effective sample size and its lack of significance of interaction effect with genetic risk and T2D incident in previous analyses. We integrated all individual lifestyle risk to “total lifestyle factors”. In a sensitivity analyses, all lifestyle and phenotypic adiposity factors were entered in the same model, to provide a conservative estimate. All statistical analyses were conducted using R, with the cmest function from the CMAverse package, and two-sided *P* < .05 was considered statistically significant.

## Results

### Study population characteristics

**Figure 1.**
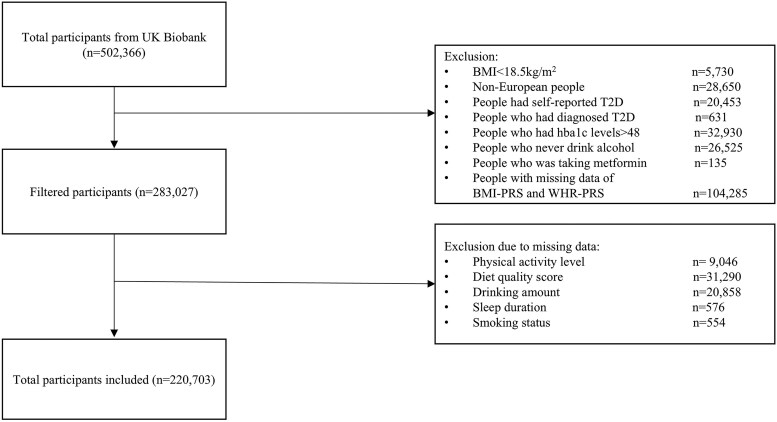
Participants flowchart.

The study population comprised *n* = 220 703 UK Biobank participants (Figure 1), with a mean age of 58 years, of whom 117 851 (53.4%) were female. Participants with higher BMI-PRS and WHR-PRS scores were less deprived and more likely to be female on average (Table 1). Overall, 46 142 (20.9%) participants had low levels of physical activity, 64 952 (29.4%) had poor quality diets, 53 710 (24.3%) had abnormal sleep duration, 102 430 (46.4%) had high alcohol consumption, 99 858 (45.2%) were ever smokers, 46 976 (21.3%) were obese, and 104 464 (47.3%) had central obesity. Of the 101 439 participants with dietary energy information, 39 313 (38.8%) had high energy intake. During a median follow-up of 8.7 years (IQR 6.12-11.42 years), 7949 participants were diagnosed with T2D by the endpoint. There were no significant interactions between sex and either BMI-PRS (*P* = .989) or WHR-PRS (*P* = .838) in relation to incident T2D. Therefore, sex stratification was not required.

**Table 1.** Participant characteristics by BMI-PRS and WHR-PRS categories (measured by *z*-score and presented by each SD).

Phenotype	BMI-PRS < −1 *n* = 36 070	BMI-PRS −1 to 0 *n* = 75 611	BMI-PRS 0 to 1 *n* = 75 255	BMI-PRS > 1 *n* = 33 767	*P*-value	WHR-PRS < −1 *n* = 35 826	WHR-PRS −1 to 0 *n* = 76 080	WHR-PRS 0 to 1 *n* = 74 714	WHR-PRS > 1 *n* = 34 083	*P*-value
Age, years, median (IQR)	58 (51, 63)	58 (50, 63)	58 (50, 63)	58 (50, 63)	<.001	58 (51, 63)	58 (50, 63)	58 (50, 63)	57 (50, 63)	<.001
Deprivation index, median (IQR)	−2.52 (−3.85, −0.34)	−2.49 (−3.82, −0.27)	−2.44 (−3.80, −0.17)	−2.40 (−3.75, −0.10)	.972	−2.52 (−3.86, −0.38)	−2.50 (−3.83, −0.29)	−2.44 (−3.79, −0.17)	−2.36 (−3.74, −0.02)	.969
Sex										
Female	19 329 (54%)	40 435 (53%)	40 252 (53%)	17 835 (53%)	.137	19 078 (53%)	40 592 (53%)	39 908 (53%)	18 273 (54%)	.799
Male	16 741 (46%)	35 176 (47%)	35 003 (47%)	15 932 (47%)		16 748 (47%)	35 488 (47%)	34 806 (47%)	15 810 (46%)	
Low physical activity level	7448 (21%)	15 568 (21%)	15 799 (21%)	7327 (22%)	<.001	7260 (20%)	15 769 (21%)	15 839 (21%)	7274 (21%)	<.001
Low diet quality	10 687 (30%)	22 293 (29%)	22 011 (29%)	9961 (29%)	.560	9911 (28%)	22 087 (29%)	22 463 (30%)	10 491 (31%)	<.001
High dietary energy intake	7018 (41%)	13 687 (39%)	13 041 (38%)	5567 (37%)	<.001	6944 (40%)	13 856 (39%)	12 915 (38%)	5598 (38%)	.021
Abnormal sleep duration	8412 (23%)	18 026 (24%)	18 536 (25%)	8736 (26%)	<.001	8154 (23%)	18 216 (24%)	18 544 (25%)	8796 (26%)	<.001
High alcohol consumption	16 794 (47%)	35 237 (47%)	34 918 (46%)	15 481 (46%)	.123	16 474 (46%)	35 002 (46%)	34 964 (47%)	15 990 (47%)	.001
Ever smoker	15 734 (44%)	33 932 (45%)	34 351 (46%)	15 841 (47%)	<.001	15 560 (43%)	34 003 (45%)	34 464 (46%)	15 831 (46%)	<.001
Obese	3518 (9.8%)	12 797 (17%)	18 579 (25%)	12 082 (36%)	<.001	5504 (15%)	14 756 (19%)	17 430 (23%)	9286 (27%)	<.001
Central obese	14 904 (41%)	34 447 (46%)	36 968 (49%)	18 145 (54%)	<.001	13 801 (39%)	34 004 (45%)	37 456 (50%)	19 203 (56%)	<.001

Numbers presented are the median (IQR) for continuous variables and the numbers (per cent) for categorical variables (*n* = 220 703). Ranges for the BMI-PRS and WHR-PRS are as follows: Q1, < −1 SD; Q2, −1 to 0 SD; Q3, 0-1 SD; Q4, >1 SD.

Abbreviations: BMI, body mass index; WHR, waist–hip ratio; PRS, polygenic risk scores.

### Mediation roles of lifestyle factors

Increased BMI-PRS and WHR-PRS were associated with higher odds of low physical activity level, abnormal sleep duration, ever smoking, and adiposity, whilst WHR-PRS was associated with higher-quality diet and higher alcohol intake. Conversely, higher BMI-PRS and WHR-PRS were associated with lower odds of high dietary energy (Figures S1 and S2).

All unhealthy lifestyle habits and adiposity factors were significantly associated with higher risk of T2D, apart from high alcohol consumption, which was significantly associated with lower risk of T2D, and high dietary energy intake which for which there was no significant association. Adiposity was associated with a nearly 4-fold risk of T2D compared with all other lifestyle factors (Figure S3).

Both BMI-PRS and WHR-PRS were significantly associated with the risk of T2D (Figures S4 and S5). Following adjustment for lifestyle risk factors, the effect sizes were not attenuated and the associations between both PRS scores and the risk of T2D remained statistically significant. Adjustment for adiposity, especially obesity, lowered the effect estimate of the associations between both BMI-PRS/WHR-PRS and incident T2D (Figures S4 and S5).

### Interactions between PRSs and lifestyle

**Figure 2.**
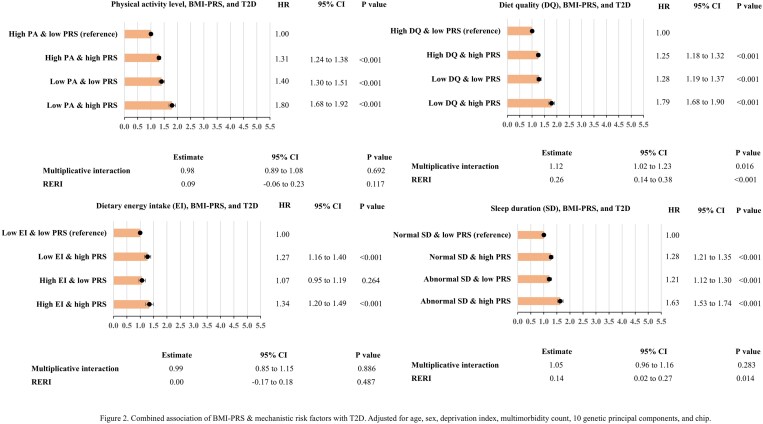
Combined association of BMI-PRS and lifestyle risk factors (low physical activity level, low diet quality, high dietary energy intake, and abnormal sleep duration) with T2D. Adjusted for age, sex, deprivation index, multimorbidity count, 10 genetic principal components, and chip. BMI-PRS, body mass index polygenic risk; T2D, type 2 diabetes.

**Figure 3.**
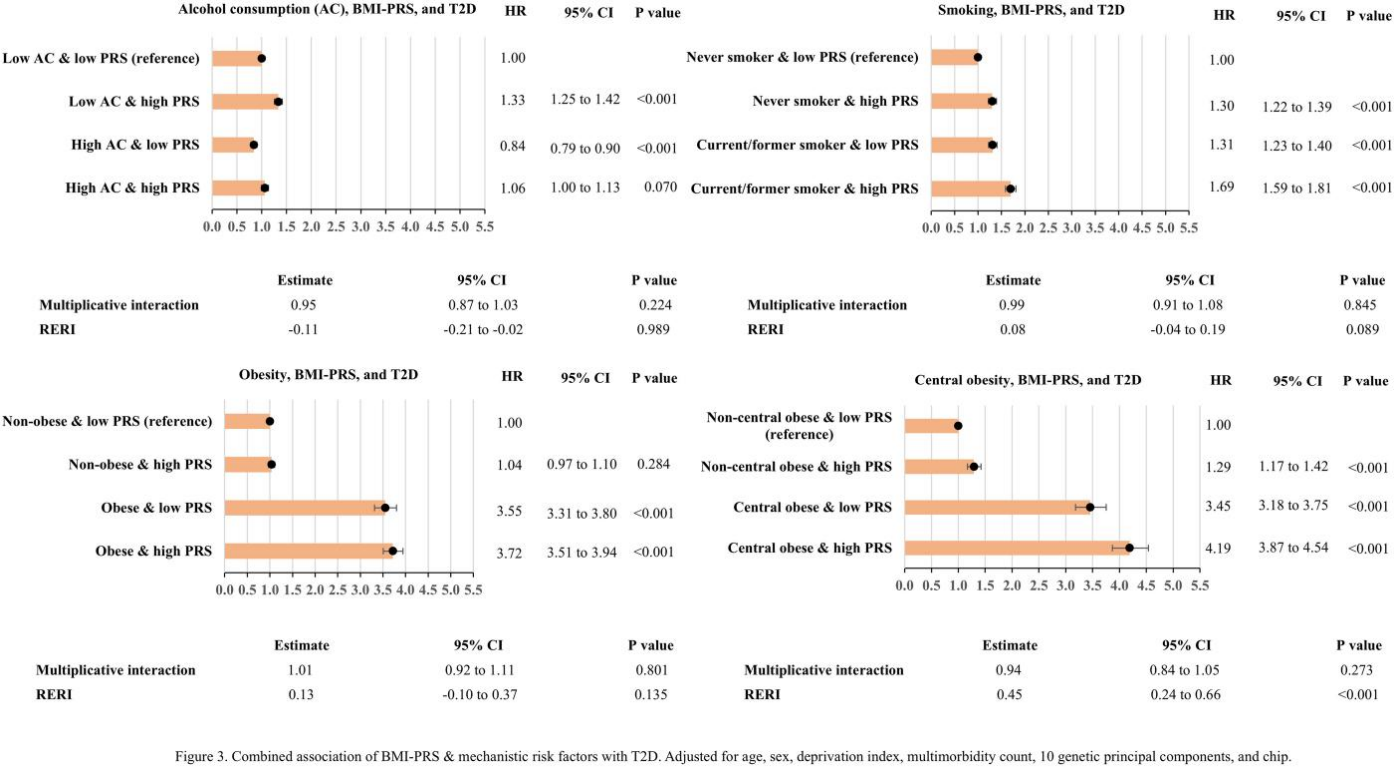
Combined association of BMI-PRS and lifestyle/adiposity risk factors high alcohol intake, current/former smoking status, obese condition, and central obesity condition) with T2D. Adjusted for age, sex, deprivation index, multimorbidity count, 10 genetic principal components, and chip. BMI-PRS, body mass index polygenic risk; T2D, type 2 diabetes.

Figures 2 and 3 present the associations between different combinations of BMI-PRS and lifestyle/phenotypic adiposity factors and incident T2D. Compared with the reference group (namely combination of T2D incident, low PRS, and healthy lifestyle pattern/non-phenotypic adiposity), there was no significantly increased risk of T2D among people with low genetic predisposition to obesity and high energy intake, and those with high genetic predisposition to obesity and high alcohol consumption, and non-phenotypic obesity (*P* > .05). There were non-significant additive interactions between BMI-PRS and all lifestyle/mediating factors except for diet quality, sleep duration, and central obesity. The only significant multiplicative interaction was between BMI-PRS and diet quality.

The results were similar for WHR-PRS and the risk of T2D (Figures S6 and S7). Phenotypic adiposity was associated with nearly 4-fold increased risk of T2D (Figures S6 and S7). Whilst all of the lifestyle/phenotypic adiposity factors were individually associated with incident T2D, people with high energy intake were not at significantly increased risk of T2D in the absence of genetic predisposition to central obesity compared to the reference group. The non-significant additive interaction was between WHR-PRS and physical activity level, and alcohol intake. None of multiplicative interactions was significant.

### Combined interaction and mediation effects

**Figure 4.**
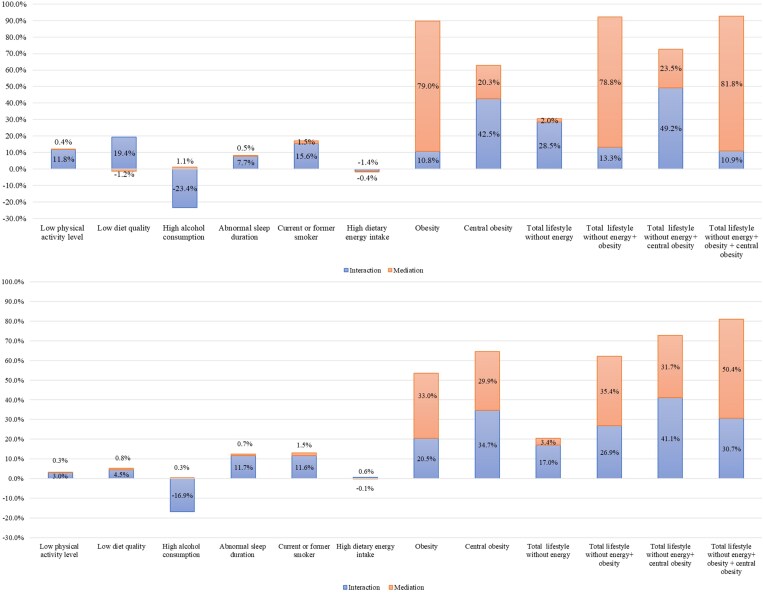
The proportion of excess risk due to BMI-PRS (top)/WHR-PRS (bottom) is attributable to the interaction and mediation of lifestyle/adiposity risk. Estimated from 4-way decomposition analysis. Adjusted for age, deprivation index, multimorbidity count, 10 genetic principal components, and chip. BMI-PRS, body mass index polygenic risk; WHR-PRS, waist-hip ratio polygenic risk.

Figure 4 summarizes the overall contribution of each individual lifestyle/phenotypic adiposity risk factor, through interaction and mediation, to the association between BMI-PRS and incident T2D, as well as the contribution of total lifestyle factors combined with phenotypic adiposity. All interactions association effects were statistically significant (*P* < .05) except for the interaction between BMI-PRS and T2D risk with high dietary energy intake, abnormal sleep duration, and phenotypic obesity, respectively, and the interaction of WHR-PRS and risk of T2D with low physical activity level, low diet quality, current or former smoker, and high energy intake.

All lifestyle and phenotypic adiposity factors were significantly (*P* < .05) mediating the relationship between BMI-PRS and the risk of T2D, except for low physical activity level and low diet quality (*P* > .05). The association between WHR-PRS and T2D was mediated by all variables except for low physical activity level, high alcohol consumption, and high energy intake. We excluded total energy intake from total lifestyle effect as it was not significant for the interaction effect in both BMI-PRS (*P* = .84) and WHR-PRS (*P* = .97), and it would largely shrink the participants sample size.

Overall, 92.7% of the association between BMI-PRS and incident T2D could be explained by all lifestyle factors and phenotypic adiposity combined (81.8% by them operating as mediators of the association and 10.9% by them modifying the effect of BMI-PRS). 30.5% of the association was due to lifestyle specifically (2.0% via mediation and 28.5% via effect modification). All the lifestyle risks studied, low physical activity level, low diet quality, and current or former smoking status individually contributed 15%-20% to interaction effect, whilst energy intake yielded the smallest contribution via either mechanism (Figure 4).

A total of 81.7% of the association between WHR-PRS and incident T2D could be explained by all lifestyle factors plus phenotypic adiposity combined (50.4% by them operating as mediators and 30.7% by them modifying the effect of WHR-PRS). A total of 20.4% of the WHR-PRS/T2D association was due to lifestyle specifically (3.4% via mediation and 17.0% via effect modification). Abnormal sleep duration and current or former status contributed around equally 13% to interaction effect, low physical activity level and low diet quality contributed relatively lower (3%-6%), and high energy intake showed the smallest effect (Figure 4).

## Discussion

This study confirmed an association between genetic predisposition to adiposity and development of T2D. We also demonstrated that whilst phenotypic adiposity explains most of this (genetic/T2D) association, modifiable lifestyle factors play a significant role. In our population cohort, individuals with a higher genetic predisposition to adiposity also tended to have lower total energy or alcohol intake. This may reflect vertical pleiotropy, where the same genetic factors that predispose to adiposity also influence lifestyle behaviours—leading people to potentially restrict their calories or alcohol consumption. Alternatively, potentially knowing they are more prone to weight gain, they may consciously limit alcohol as part of weight management, often reinforced by healthcare advice. Moreover, there is J-shaped relationship between alcohol consumption and T2D. Whilst extremely low and very high intakes are usually associated with increased risk, moderate consumption can appear protective.^34^ Our definition of “high” alcohol intake (>14 units/week) may still fall within the “moderate” or “moderately high” range in other studies, which could underlie its association with a lower observed risk of T2D in our analyses. Phenotypic obesity accounted for around 90% of the association between genetic predisposition to obesity and T2D risk, and phenotypic central obesity explained 63%. However, around 30% of the associations could be explained by a range of modifiable lifestyle factors, working either directly or via an increased risk of adiposity. For the association between genetic predisposition to central obesity and T2D risk, nearly 55%-65% of it was accounted for by phenotypic adiposity. Nearly 20% of the association was explained by total lifestyle factors. Therefore, the relative contribution of lifestyle to increased risk of T2D was greatest among those with a genetic predisposition to adiposity. Genetic predisposition to adiposity does not inevitably lead to increased risk of T2D—the association between genetic predisposition and phenotypic adiposity is strongly dependent on lifestyle factors.

Previous studies have investigated the role of genetic predisposition to T2D using specific T2D SNPs.^2,9,35,36^ There is a paucity of evidence relating to the role of genetic predisposition to adiposity and the extent to which its association with T2D might be mitigated. Our results are consistent with a previous study which demonstrated that people with high genetic predisposition to adiposity had a significant tendency towards worse lifestyle patterns of low physical activity level, unhealthy sleep duration, ever smoking status, and adiposity-associated conditions,^8^ and these unfavourable lifestyle factors are associated with higher risk of T2D.^37^ Few studies have focused on the mediation effect of lifestyle/adiposity status on the relationship between genetic variants and T2D.

Our study has several strengths. First, UK Biobank is a large prospective study with long extent of follow-up in the large population cohort. Our exclusion criteria of people who were underweight, never drink alcohol as well as people who were diagnosed T2D at baseline and had >48 (mmol/mol) HbA1c level lower the risk of reverse causation impacting our findings. Furthermore, the exposure of genetic predisposition is inborn, which is not normally changed by environmental confounders. In addition, our study design investigated 2 different measures of adiposity as exposures and mediators, BMI-PRS/BMI as a measure of general obesity and WHR-PRS/WHR as a measure of central obesity, meaning that multiple adiposity types were investigated. A wide range of lifestyle factors were included, as well as whether they were associated with genetic predisposition to adiposity and T2D independently of each other, and whether they mediated these associations. Finally, for analyses, the novel statistical method of interaction and 4-way decomposition analyses (for combining interaction and mediation) provide good insight into how much of the effect is attributable to mediation, interaction, both mediation and interaction together, or neither.

Our study has limitations. First, within UK Biobank, our findings could not be applied to other ethnic groups, and White British participants were the only sub-population sufficiently powered to study genetic interactions and mediations.^38^ Second, although we adjusted for potential confounders such as age, sex, and socioeconomic status, residual confounding is still possible in an observational study. Lifestyle factors were self-reported and potentially subject to measurement error and reporting bias,^39^ and adiposity and lifestyle variables were assessed at a specific single moment in time which may change over time. Future research may validate results for different lifestyle profiles in a wider range of demographics and explore age-stratified analyses or other subgroup evaluations—provided there is sufficient power to support the generalizability of our results. Finally, whilst we investigated a range of lifestyle factors but could not identify weight type (when it is increasing because of increased lean body mass), future research needs to focus on identifying specific different weight type to broaden our understanding of gene–lifestyle interactions and mediation.^40^

In summary, our study shows that whilst genetic predisposition to adiposity is associated with increased risk of T2D, a significant proportion of the association can be explained by modifiable lifestyle factors operating either directly or by contributing to obesity or central obesity. Furthermore, the contribution of lifestyle factors is highest in those with high genetic predisposition. Therefore, higher risk of T2D among people genetically predisposed to adiposity is not inevitable and could be reduced via lifestyle modification.

## Supplementary Material

lvaf084_Supplementary_Data

## Data Availability

Data used for all analyses are from UK Biobank. Data are available via application to the UK Biobank (http://www.ukbiobank.ac.uk).
