# Supplementary material for: Genetic predisposition to adiposity, and type 2 diabetes: the role of lifestyle and phenotypic adiposity
Source: Eur J Endocrinol. 2025 May 2;192(5):549–57. doi: 10.1093/ejendo/lvaf084 (PMC12056655; doi:10.1093/ejendo/lvaf084)
Supplement: lvaf084_Supplementary_Data [file lvaf084_supplementary_data.zip › eje-25-0105-File007.pptx]

## Slide 1
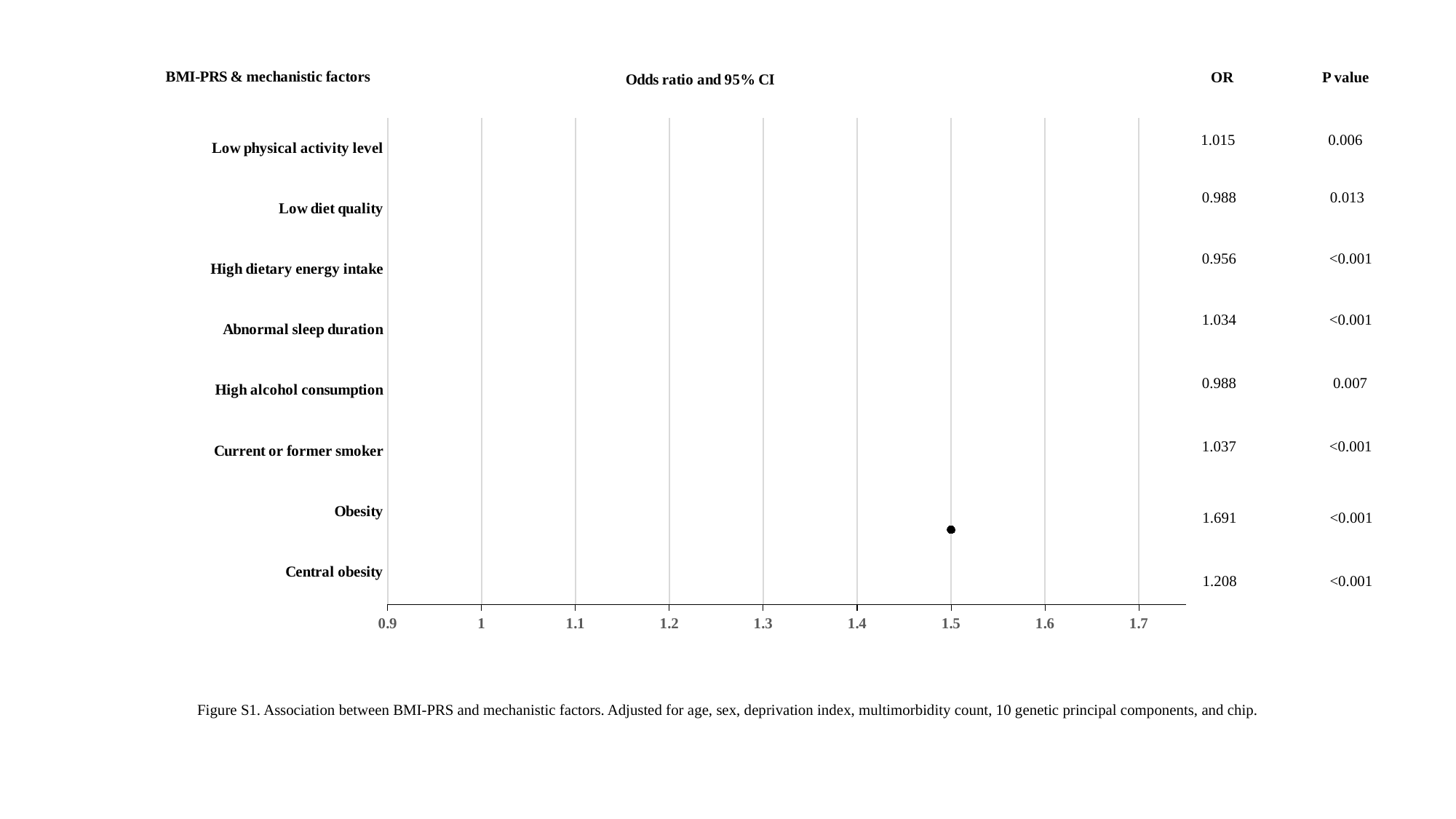

OR
P value
### Chart: Odds ratio and 95% CI
| Category | Odds Ratio | |
|---|---|---|
| Central obesity | 1.208 | 0.5 |
| Obesity | 1.691 | 1.5 |
| Current or former smoker | 1.037 | 2.5 |
| High alcohol consumption | 0.988 | 3.5 |
| Abnormal sleep duration | 1.034 | 4.5 |
| High dietary energy intake | 0.956 | 5.5 |
| Low diet quality | 0.988 | 6.5 |
| Low physical activity level | 1.015 | 7.5 |1.015
0.006
0.988
0.013
0.956
<0.001
1.034
<0.001
0.988
 0.007
1.037
<0.001
1.691
<0.001
1.208
<0.001
Figure S1. Association between BMI-PRS and mechanistic factors. Adjusted for age, sex, deprivation index, multimorbidity count, 10 genetic principal components, and chip.

## Slide 2
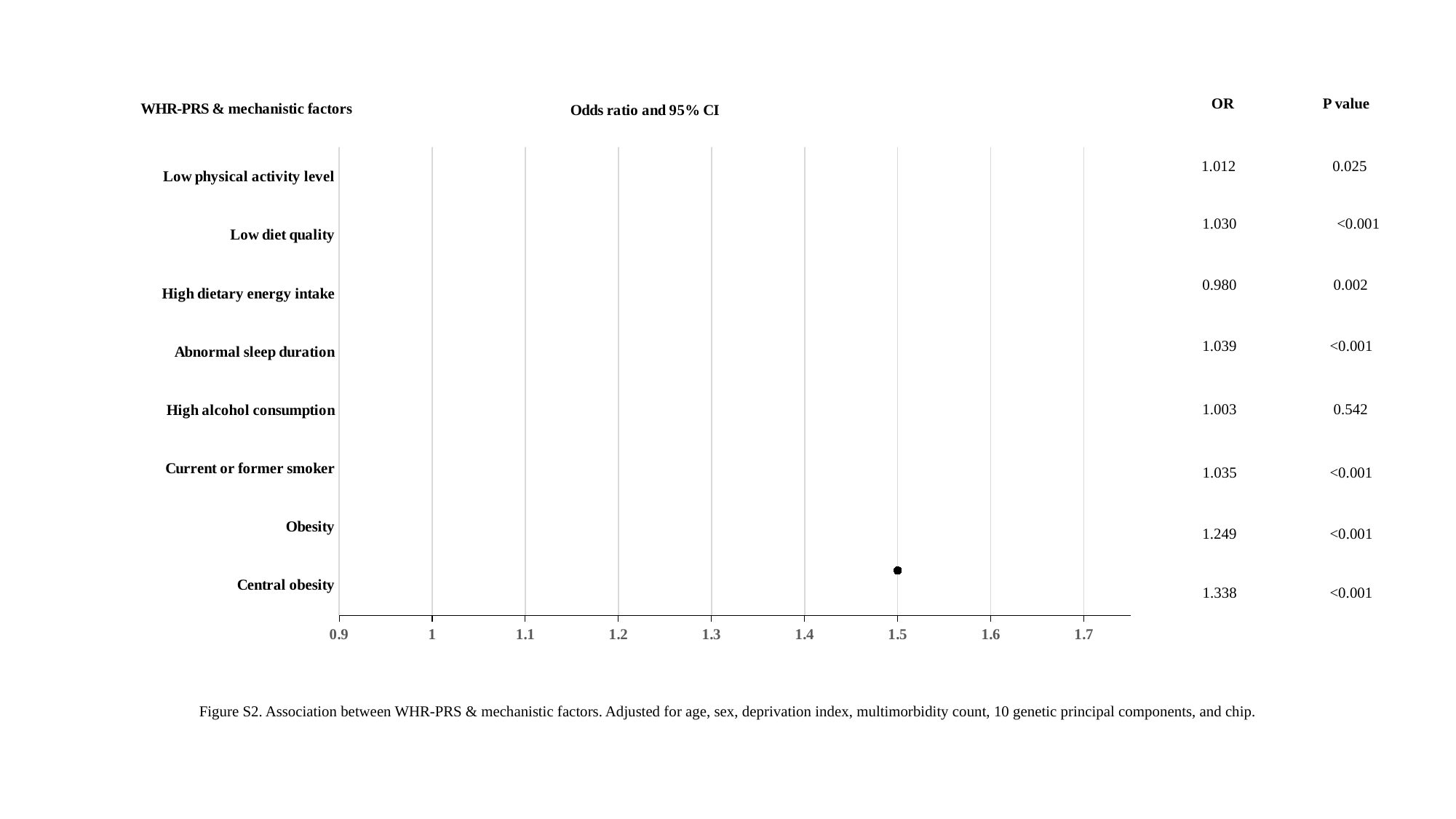

OR
P value
### Chart: Odds ratio and 95% CI
| Category | Odds Ratio | |
|---|---|---|
| Central obesity | 1.338 | 0.5 |
| Obesity | 1.249 | 1.5 |
| Current or former smoker | 1.035 | 2.5 |
| High alcohol consumption | 1.003 | 3.5 |
| Abnormal sleep duration | 1.039 | 4.5 |
| High dietary energy intake | 0.98 | 5.5 |
| Low diet quality | 1.03 | 6.5 |
| Low physical activity level | 1.012 | 7.5 |1.012
 0.025
1.030
<0.001
0.980
 0.002
1.039
<0.001
1.003
 0.542
1.035
<0.001
1.249
<0.001
1.338
<0.001
Figure S2. Association between WHR-PRS & mechanistic factors. Adjusted for age, sex, deprivation index, multimorbidity count, 10 genetic principal components, and chip.

## Slide 3
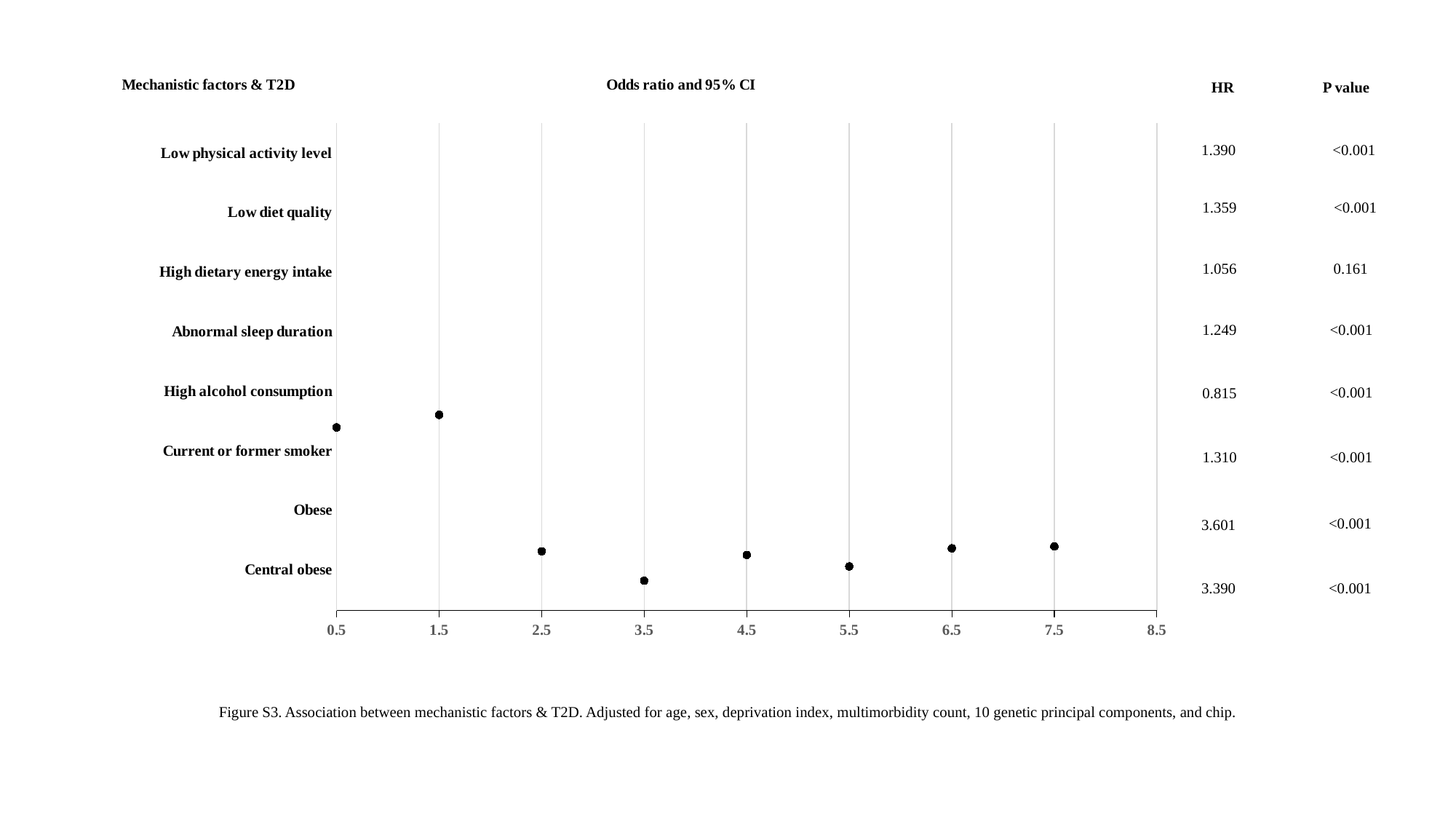

### Chart: Odds ratio and 95% CI
| Category | Odds Ratio | |
|---|---|---|
| Central obese | 3.3899 | 0.5 |
| Obese | 3.6007 | 1.5 |
| Current or former smoker | 1.31 | 2.5 |
| High alcohol consumption | 0.8149 | 3.5 |
| Abnormal sleep duration | 1.2488 | 4.5 |
| High dietary energy intake | 1.0559 | 5.5 |
| Low diet quality | 1.3594 | 6.5 |
| Low physical activity level | 1.3901 | 7.5 |HR
P value
1.390
 <0.001
1.359
<0.001
1.056
 0.161
1.249
<0.001
<0.001
0.815
1.310
<0.001
<0.001
3.601
3.390
<0.001
Figure S3. Association between mechanistic factors & T2D. Adjusted for age, sex, deprivation index, multimorbidity count, 10 genetic principal components, and chip.

## Slide 4
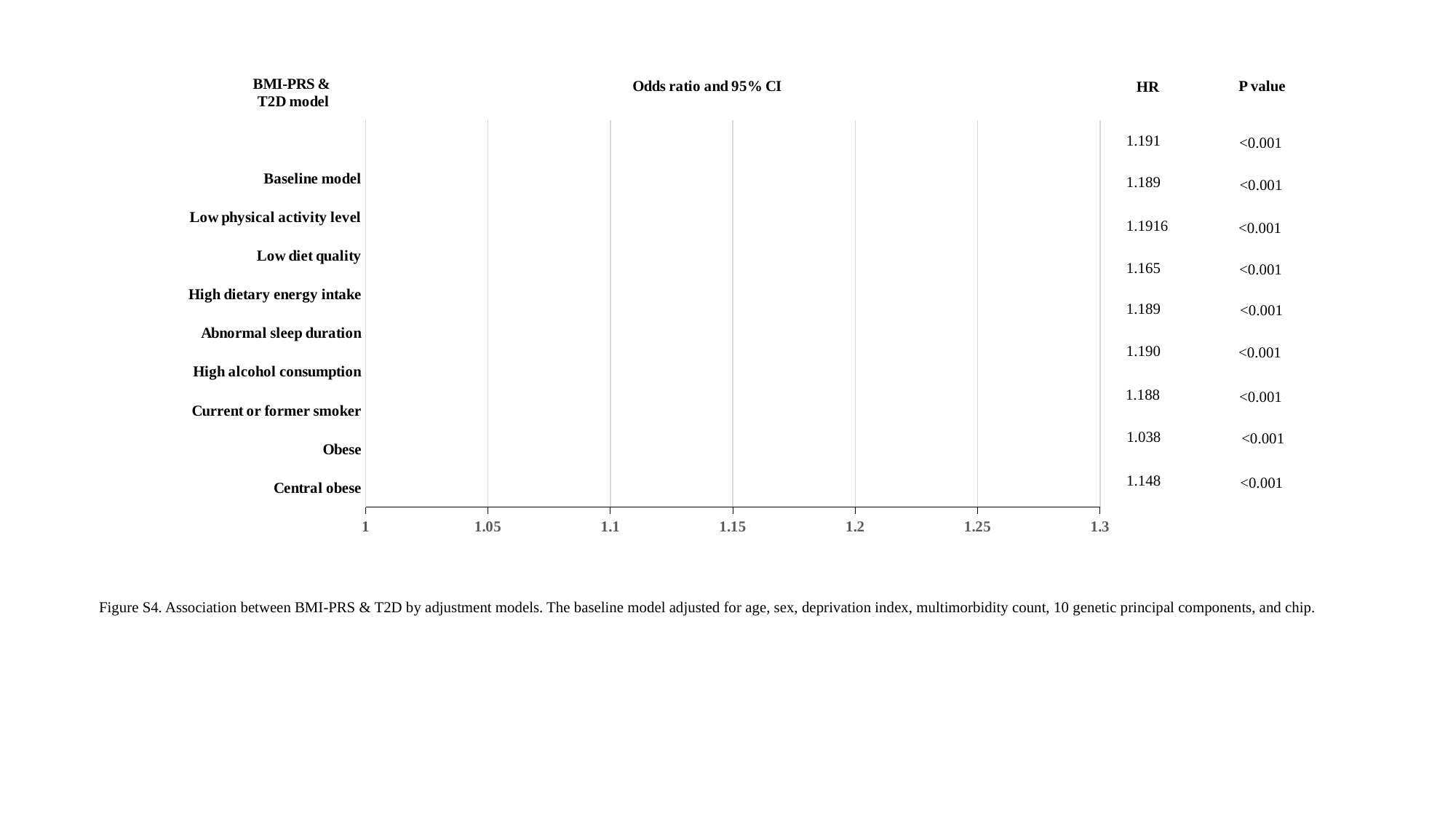

### Chart: Odds ratio and 95% CI
| Category | Odds Ratio | |
|---|---|---|
| Central obese | 1.148 | 0.5 |
| Obese | 1.0377 | 1.5 |
| Current or former smoker | 1.1875 | 2.5 |
| High alcohol consumption | 1.1895 | 3.5 |
| Abnormal sleep duration | 1.1892 | 4.5 |
| High dietary energy intake | 1.1652 | 5.5 |
| Low diet quality | 1.1916 | 6.5 |
| Low physical activity level | 1.1892 | 7.5 |
| Baseline model | 1.1906 | 8.5 |P value
HR
1.191
<0.001
1.189
<0.001
1.1916
<0.001
1.165
<0.001
1.189
<0.001
1.190
<0.001
1.188
<0.001
1.038
<0.001
1.148
<0.001
Figure S4. Association between BMI-PRS & T2D by adjustment models. The baseline model adjusted for age, sex, deprivation index, multimorbidity count, 10 genetic principal components, and chip.

## Slide 5
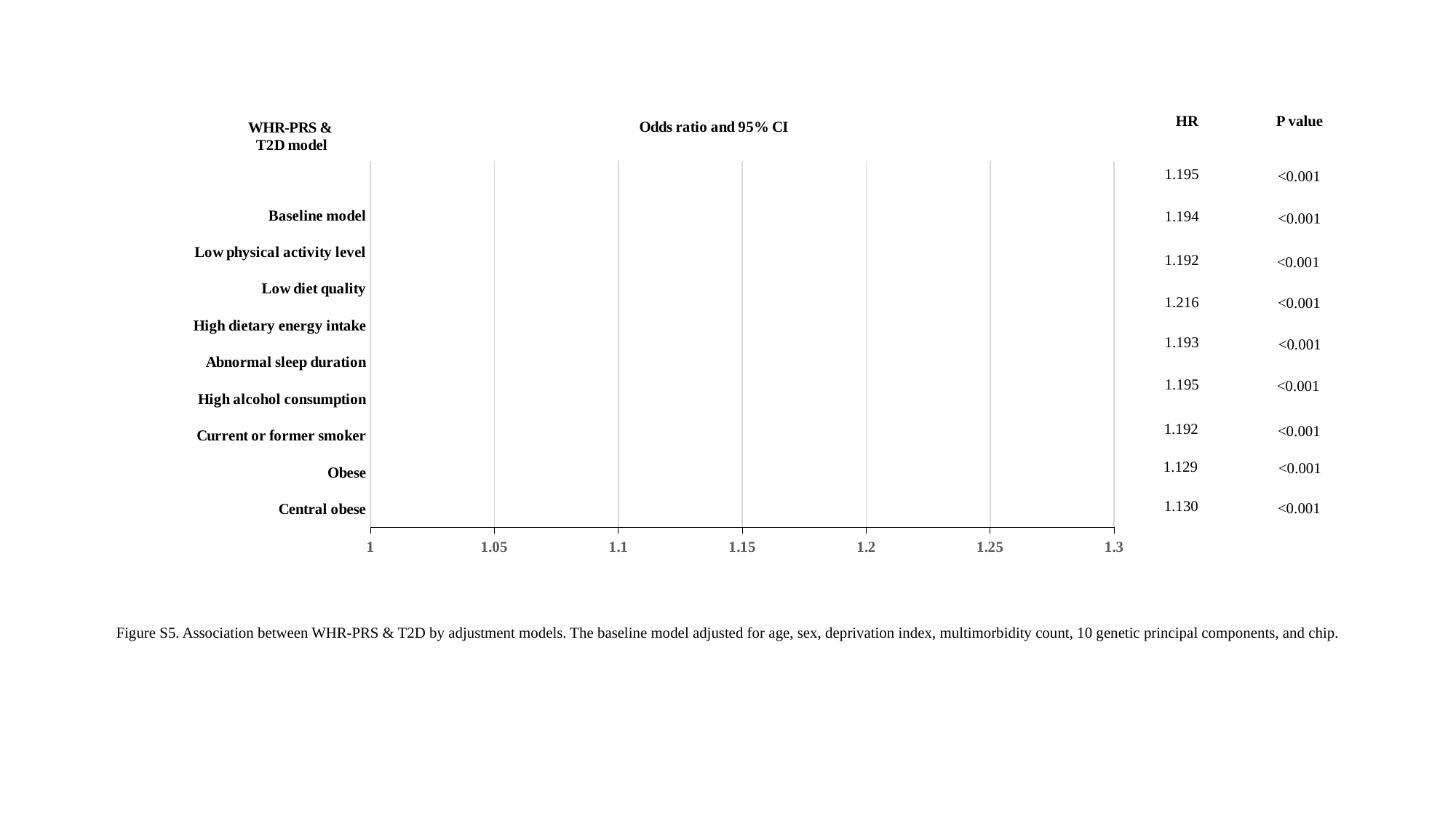

HR
P value
### Chart: Odds ratio and 95% CI
| Category | Odds Ratio | |
|---|---|---|
| Central obese | 1.1303 | 0.5 |
| Obese | 1.1292 | 1.5 |
| Current or former smoker | 1.1916 | 2.5 |
| High alcohol consumption | 1.1951 | 3.5 |
| Abnormal sleep duration | 1.1929 | 4.5 |
| High dietary energy intake | 1.2164 | 5.5 |
| Low diet quality | 1.192 | 6.5 |
| Low physical activity level | 1.1941 | 7.5 |
| Baseline model | 1.1949 | 8.5 |1.195
<0.001
1.194
<0.001
1.192
<0.001
1.216
<0.001
1.193
<0.001
1.195
<0.001
1.192
<0.001
1.129
<0.001
1.130
<0.001
Figure S5. Association between WHR-PRS & T2D by adjustment models. The baseline model adjusted for age, sex, deprivation index, multimorbidity count, 10 genetic principal components, and chip.

## Slide 6
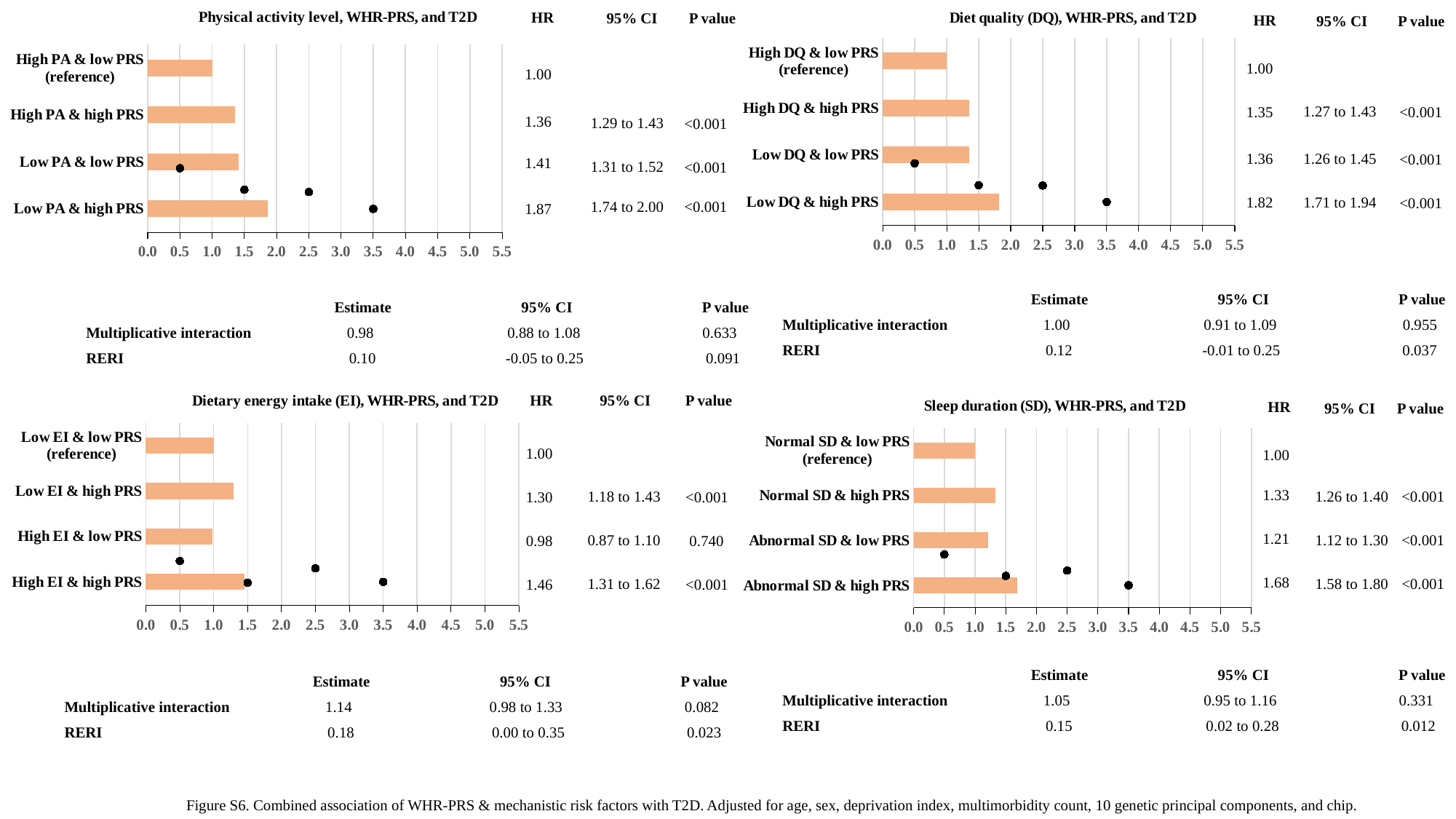

### Chart: Diet quality (DQ), WHR-PRS, and T2D
| Category | OR / interaction | |
|---|---|---|
| Low DQ & high PRS | 1.82039066577161 | 0.5 |
| Low DQ & low PRS | 1.3551919871011 | 1.5 |
| High DQ & high PRS | 1.3468572292423 | 2.5 |
| High DQ & low PRS (reference) | 1.0 | 3.5 |
### Chart: Physical activity level, WHR-PRS, and T2D
| Category | OR / interaction | |
|---|---|---|
| Low PA & high PRS | 1.86599275511497 | 0.5 |
| Low PA & low PRS | 1.40711016482537 | 1.5 |
| High PA & high PRS | 1.35838674826559 | 2.5 |
| High PA & low PRS (reference) | 1.0 | 3.5 |HR
95% CI
P value
HR
95% CI
P value
<0.001
<0.001
<0.001
1.27 to 1.43
1.26 to 1.45
1.71 to 1.94
1.00
1.35
1.36
1.82
<0.001
<0.001
<0.001
1.29 to 1.43
1.31 to 1.52
1.74 to 2.00
1.00
1.36
1.41
1.87
 Estimate 95% CI P value
Multiplicative interaction 1.00 0.91 to 1.09 0.955
RERI 0.12 -0.01 to 0.25 0.037
 Estimate 95% CI P value
Multiplicative interaction 0.98 0.88 to 1.08 0.633
RERI 0.10 -0.05 to 0.25 0.091
### Chart: Dietary energy intake (EI), WHR-PRS, and T2D
| Category | OR / interaction | |
|---|---|---|
| High EI & high PRS | 1.45783287775682 | 0.5 |
| High EI & low PRS | 0.980800694386401 | 1.5 |
| Low EI & high PRS | 1.29864808534524 | 2.5 |
| Low EI & low PRS (reference) | 1.0 | 3.5 |HR
95% CI
P value
### Chart: Sleep duration (SD), WHR-PRS, and T2D
| Category | OR / interaction | |
|---|---|---|
| Abnormal SD & high PRS | 1.68452539775278 | 0.5 |
| Abnormal SD & low PRS | 1.20904995496225 | 1.5 |
| Normal SD & high PRS | 1.32802038972735 | 2.5 |
| Normal SD & low PRS (reference) | 1.0 | 3.5 |HR
P value
95% CI
<0.001
<0.001
<0.001
<0.001
 0.740
<0.001
1.26 to 1.40
1.12 to 1.30
1.58 to 1.80
1.18 to 1.43
0.87 to 1.10
1.31 to 1.62
1.00
1.30
0.98
1.46
1.00
1.33
1.21
1.68
 Estimate 95% CI P value
Multiplicative interaction 1.05 0.95 to 1.16 0.331
RERI 0.15 0.02 to 0.28 0.012
 Estimate 95% CI P value
Multiplicative interaction 1.14 0.98 to 1.33 0.082
RERI 0.18 0.00 to 0.35 0.023
Figure S6. Combined association of WHR-PRS & mechanistic risk factors with T2D. Adjusted for age, sex, deprivation index, multimorbidity count, 10 genetic principal components, and chip.

## Slide 7
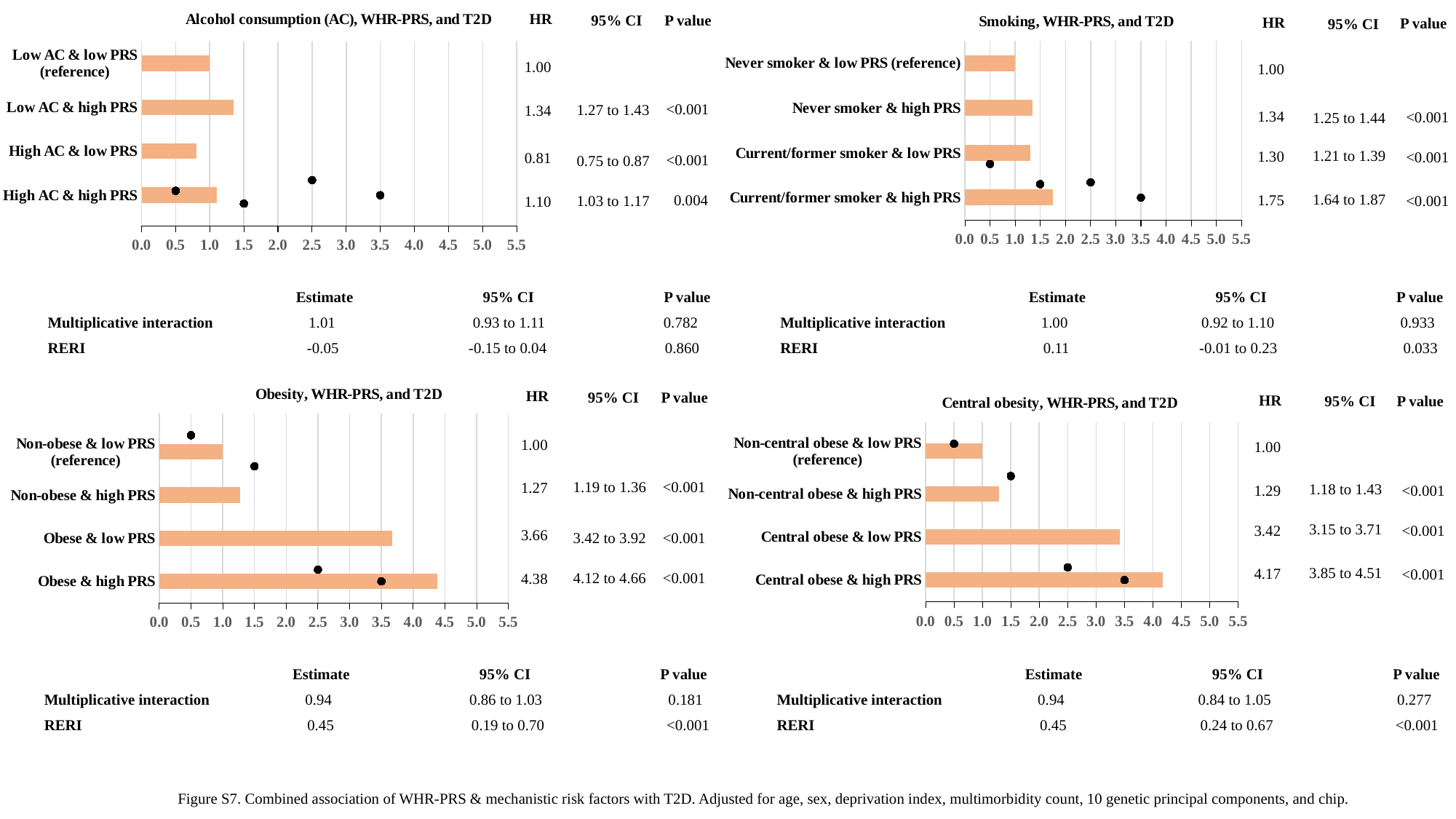

### Chart: Smoking, WHR-PRS, and T2D
| Category | OR / interaction | |
|---|---|---|
| Current/former smoker & high PRS | 1.74980198255179 | 0.5 |
| Current/former smoker & low PRS | 1.2995837680216 | 1.5 |
| Never smoker & high PRS | 1.34126195960546 | 2.5 |
| Never smoker & low PRS (reference) | 1.0 | 3.5 |
### Chart: Alcohol consumption (AC), WHR-PRS, and T2D
| Category | OR / interaction | |
|---|---|---|
| High AC & high PRS | 1.10018545839494 | 0.5 |
| High AC & low PRS | 0.808188392299165 | 1.5 |
| Low AC & high PRS | 1.34422613109616 | 2.5 |
| Low AC & low PRS (reference) | 1.0 | 3.5 |HR
95% CI
P value
HR
P value
95% CI
1.27 to 1.43
0.75 to 0.87
1.03 to 1.17
<0.001
<0.001
<0.001
1.25 to 1.44
1.21 to 1.39
1.64 to 1.87
1.00
1.34
0.81
1.10
1.00
1.34
1.30
1.75
<0.001
<0.001
 0.004
 Estimate 95% CI P value
Multiplicative interaction 1.00 0.92 to 1.10 0.933
RERI 0.11 -0.01 to 0.23 0.033
 Estimate 95% CI P value
Multiplicative interaction 1.01 0.93 to 1.11 0.782
RERI -0.05 -0.15 to 0.04 0.860
### Chart: Obesity, WHR-PRS, and T2D
| Category | OR / interaction | |
|---|---|---|
| Obese & high PRS | 4.38202211848201 | 0.5 |
| Obese & low PRS | 3.66421313330165 | 1.5 |
| Non-obese & high PRS | 1.27090066114845 | 2.5 |
| Non-obese & low PRS (reference) | 1.0 | 3.5 |
### Chart: Central obesity, WHR-PRS, and T2D
| Category | OR / interaction | |
|---|---|---|
| Central obese & high PRS | 4.16788770625345 | 0.5 |
| Central obese & low PRS | 3.41888265513075 | 1.5 |
| Non-central obese & high PRS | 1.29481461082905 | 2.5 |
| Non-central obese & low PRS (reference) | 1.0 | 3.5 |HR
95% CI
P value
HR
P value
95% CI
1.19 to 1.36
3.42 to 3.92
4.12 to 4.66
<0.001
<0.001
<0.001
1.18 to 1.43
3.15 to 3.71
3.85 to 4.51
1.00
1.27
3.66
4.38
1.00
1.29
3.42
4.17
<0.001
<0.001
<0.001
 Estimate 95% CI P value
Multiplicative interaction 0.94 0.84 to 1.05 0.277
RERI 0.45 0.24 to 0.67 <0.001
 Estimate 95% CI P value
Multiplicative interaction 0.94 0.86 to 1.03 0.181
RERI 0.45 0.19 to 0.70 <0.001
Figure S7. Combined association of WHR-PRS & mechanistic risk factors with T2D. Adjusted for age, sex, deprivation index, multimorbidity count, 10 genetic principal components, and chip.
