# Supplementary material for: Genetic predisposition to adiposity, and type 2 diabetes: the role of lifestyle and phenotypic adiposity
Source: Eur J Endocrinol. 2025 May 2;192(5):549–57. doi: 10.1093/ejendo/lvaf084 (PMC12056655; doi:10.1093/ejendo/lvaf084)
Supplement: lvaf084_Supplementary_Data [file lvaf084_supplementary_data.zip › eje-25-0105-File008.docx]

**GWAS Summary Statistics**

**BMI Summary Statistics:** We obtained the GWAS summary statistics for BMI from a large meta-analysis of primarily European ancestry individuals ^1^. These data include millions of genetic variants after imputation with effect sizes.

**WHR Summary Statistics:** For WHR, we used a similarly large-scale meta-analysis of GWAS datasets focusing on individuals of European ancestry to ensure compatibility with the UK Biobank population ^2^. This study includes the loci for WHR which are enriched for association with different metabolic traits ^3^.

**Quality Control for Summary Statistics**

**Allele Matching and Filtering:** We harmonized the summary statistics to the UK Biobank reference genome build, ensuring allele frequency consistency. Variants were excluded if they had mismatched or ambiguous alleles, extremely low minor allele frequency (MAF <0.0001), or poor imputation quality. This step prevented spurious associations that can arise from unaligned reference/alternate alleles.

**LD Reference Panel and LDpred**

**LD Reference Panel:** We set aside 1,000 unrelated white British participants who are recommended in the LDpred paper ^4^ - meeting the stringent quality control criteria described in our Methods - to serve as the linkage disequilibrium (LD) reference panel. These individuals were not used in the main analyses. These participants are white British, whose self-reported sex match their genetically determined sex, who do not have purported sex chromosome aneuploidy, and who are not determined by UK Biobank to be outliers for heterozygosity.

**LD Adjustment (LDpred):** We applied LDpred in its infinitesimal mode, which assumes all variants have nonzero, normally distributed effects. The software corrects for LD among SNPs by using the reference panel to estimate local correlation structures. This yields an LD-adjusted weight for each SNP in the genome-wide panel of variants.

**Polygenic Score Computation**

**Weight Calculation:** The final result of LDpred provides a per-variant weight (adjusted effect size) reflecting that SNP’s contribution to BMI and WHR.

**Score Generation:** For each participant - excluding those in the reference panel - we computed the PRS by summing the products of genotype and the corresponding SNP weights across the retained set of variants.

By combining large-scale, high-quality GWAS summary statistics with rigorous quality control and the LDpred methodology, we aimed to generate robust, genome-wide scores reflecting individuals’ genetic predispositions to overall and central adiposity. These comprehensive steps ensure that our BMI-PRS and WHR-PRS capture relevant common genetic variation while maintaining consistency and accuracy in the UK Biobank cohort.

**Reference**

1. Locke AE, Kahali B, Berndt SI, Justice AE, Pers TH, Day FR, Powell C, Vedantam S, Buchkovich ML & Yang J. Genetic studies of body mass index yield new insights for obesity biology. *Nature* 2015 **518** 197-206.

2. Shungin D, Winkler TW, Croteau-Chonka DC, Ferreira T, Locke AE, Mägi R, Strawbridge RJ, Pers TH, Fischer K & Justice AE. New genetic loci link adipose and insulin biology to body fat distribution. *Nature* 2015 **518** 187-196.

3. Heid IM, Jackson AU, Randall JC, Winkler TW, Qi L, Steinthorsdottir V, Thorleifsson G, Zillikens MC, Speliotes EK & Mägi R. Meta-analysis identifies 13 new loci associated with waist-hip ratio and reveals sexual dimorphism in the genetic basis of fat distribution. *Nature Genetics* 2010 **42** 949-960.

4. Vilhjálmsson BJ, Yang J, Finucane HK, Gusev A, Lindström S, Ripke S, Genovese G, Loh P-R, Bhatia G & Do R. Modeling linkage disequilibrium increases accuracy of polygenic risk scores. *The american journal of human genetics* 2015 **97** 576-592.
